# JAMIR-eQTL: Japanese genome-wide identification of microRNA expression quantitative trait loci across dementia types

**DOI:** 10.1093/database/baab072

**Published:** 2021-11-03

**Authors:** Shintaro Akiyama, Sayuri Higaki, Takahiro Ochiya, Kouichi Ozaki, Shumpei Niida, Daichi Shigemizu

**Affiliations:** Medical Genome Center, Research Institute, National Center for Geriatrics and Gerontology, Aichi 474-8511, Japan; Clinical Research Center, National Hospital Organization Nagoya Medical Center, Aichi 460-0001, Japan; Institute of Medical Science, Tokyo Medical University, Tokyo 160-8402, Japan; Medical Genome Center, Research Institute, National Center for Geriatrics and Gerontology, Aichi 474-8511, Japan; Center for Integrative Medical Sciences, RIKEN Center for Integrative Medical Sciences, Yokohama 230-0045, Japan; Medical Genome Center, Research Institute, National Center for Geriatrics and Gerontology, Aichi 474-8511, Japan; Medical Genome Center, Research Institute, National Center for Geriatrics and Gerontology, Aichi 474-8511, Japan; Center for Integrative Medical Sciences, RIKEN Center for Integrative Medical Sciences, Yokohama 230-0045, Japan; Medical Research Institute, Tokyo Medical and Dental University (TMDU), Tokyo 113-8510, Japan

## Abstract

MicroRNAs (miRNAs) are small non-coding RNAs shown to regulate gene expression by binding to complementary transcripts. Genetic variants, including single-nucleotide polymorphisms and short insertions/deletions, contribute to traits and diseases by influencing miRNA expression. However, the association between genetic variation and miRNA expression remains to be elucidated. Here, by using genotype data and miRNA expression data from 3448 Japanese serum samples, we developed a computational pipeline to systematically identify genome-wide miRNA expression quantitative trait loci (miR-eQTLs). Not only did we identify a total of 2487 *cis*-miR-eQTLs and 3 155 773 *trans*-miR-eQTLs at a false discovery rate of <0.05 in six dementia types (Alzheimer’s disease, dementia with Lewy bodies, vascular dementia, frontotemporal lobar degeneration, normal-pressure hydrocephalus and mild cognitive impairment) and all samples, including those from patients with other types of dementia, but also we examined the commonality and specificity of miR-eQTLs among dementia types. To enable data searching and downloading of these *cis*- and *trans*-eQTLs, we developed a user-friendly database named JAMIR-eQTL, publicly available at https://www.jamir-eqtl.org/. This is the first miR-eQTL database designed for dementia types. Our integrative and comprehensive resource will contribute to understanding the genetic basis of miRNA expression as well as to the discovery of deleterious mutations, particularly in dementia studies.

**Database URL**: https://www.jamir-eqtl.org/

## Introduction

MicroRNAs (miRNAs) are small non-coding RNA (ncRNA) molecules of ∼22 nucleotides that play key roles in the post-transcriptional regulation of gene expression and control of biological processes in development, differentiation, growth, apoptosis and metabolism. miRNAs have been the focus of recent studies as potential biomarkers of a variety of diseases, including cancers (1, 2), cardiovascular disease (3) and Alzheimer’s disease (4, 5). Many experimental techniques and computational methods have been developed to detect miRNAs (6–8), and the number of newly identified miRNAs is still increasing (9).

The development of high-throughput genotyping and massive parallel next-generation sequencing enables the genotyping of a large number of genetic variants, including single-nucleotide polymorphisms (SNPs) and short insertions/deletions (indels). Genome-wide association studies have successfully identified numerous genetic variants associated with human complex traits (10, 11) and diseases (12, 13). However, as the majority of trait- or disease-associated loci are located in non-coding regions of the genome, it remains challenging to determine their underlying basic mechanism or effect on pathogenesis (14); the targets they affect are often assigned to neighboring protein-coding genes or ncRNAs.

Expression quantitative trait locus (eQTL) analysis is a powerful approach to elucidating the biological correlations between gene expression and genetic variants and helps to explain the regulatory mechanisms underlying trait- or disease-associated loci (15, 16). Although some eQTL databases have been established, most of them have been developed for the associations between genotypes and protein-coding genes (17, 18). Only one ncRNA-related eQTL database, called ncRNA-eQTL, has been recently developed, for different cancer types (19), although a few studies of miRNA expression quantitative trait loci (miR-eQTLs) have reported the findings of analyses of a small number of samples and a small number of miRNAs (20, 21).

Here, we investigated the statistically significant associations between genetic variants and comprehensive miRNA expression by using a large number of samples representing different types of dementia. Not only did we develop a computational pipeline to systematically identify miRNA-eQTLs in different dementia types, including mild cognitive impairment, but also we constructed a user-friendly database, JAMIR-eQTL, to enable data searching and downloading of these *cis*-eQTLs and *trans*-eQTLs. Our integrative and comprehensive resource will contribute to understanding the genetic basis of miRNA expression, as well as to the discovery of deleterious mutations associated with human complex diseases, including dementia. Furthermore, since miRNAs have been reported to be associated with many diseases including cancer (19) and dementia (5, 22), the integration of other databases will contribute to the elucidation of the cross-disease interpretation of miRNAs.

## Data collection and processing

### Subjects

Serum samples from 3448 Japanese patients registered with the National Center for Geriatrics and Gerontology (NCGG) Biobank were used and represented six types of dementia [Alzheimer’s disease (AD), dementia with Lewy bodies (DLB), vascular dementia (VaD), frontotemporal lobar degeneration (FTLD), normal-pressure hydrocephalus (NPH) and mild cognitive impairment (MCI)] as well as ‘Other’, which included all samples not representing one of the six dementia types. The numbers of subjects were 1314 for AD, 134 for DLB, 69 for VaD, 31 for FTLD, 39 for NPH, 504 for MCI and 1357 for Other. All subjects provided written informed consent. The study was approved by the NCGG ethical committee.

### Genotyping data

**Figure 1.**
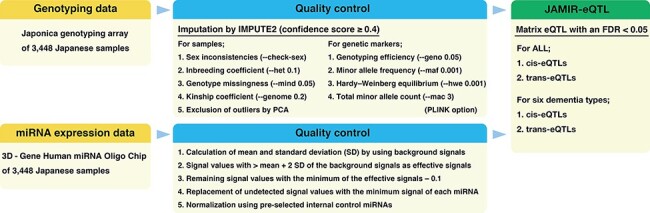
Flowchart of the JAMIR-eQTL database.

Genotyping data were downloaded from the NCGG Biobank database. Those data were obtained according to established protocols. Genomic DNA was extracted from peripheral blood leukocytes by using standard protocols and a Maxwell RSC [Rapid Sample Concentrator] Instrument (Promega). All 3448 subjects were genotyped by using the Affymetrix Japonica genotyping array (Toshiba Corporation, Tokyo, Japan) (23). Genotype imputation was performed by using IMPUTE2 (ver. 2.3.2) (24) with the 3.5K Japanese reference panel (12). We used autosomal SNPs and short indels with an imputation confidence score ≥0.4. Quality control (QC) was performed by using PLINK software (25). We applied the following QC filters to the subjects: (i) sex inconsistencies (–check-sex); (ii) inbreeding coefficient (–het 0.1); (iii) genotype missingness (–mind 0.05); (iv) kinship coefficient (–genome 0.2) and (v) exclusion of outliers from the clusters of East Asian populations in a principal component analysis that was conducted with 1000 Genomes Phase 3 data. We further applied the following QC filters to the genetic markers (SNPs and indels): (i) genotyping efficiency or call rate (–geno 0.05); (ii) minor allele frequency (–maf 0.001); (iii) Hardy–Weinberg equilibrium (–hwe 0.001) and (iv) total minor allele count (–mac 3). A total of 9 367 559 genetic markers from all 3448 subjects passed the QC criteria after imputation: 8 948 088 genetic markers from 1314 AD subjects, 6 133 945 from 134 DLB subjects, 5 448 340 from 69 VaD subjects, 4 588 639 from 31 FTLD subjects, 4 754 596 from 39 NPH subjects and 7 766 083 from 504 MCI subjects (Figure 1).

### miRNA expression data

Blood miRNA profiling data were also downloaded from the NCGG Biobank database. Comprehensive miRNA expression data were generated by using a 3D‐Gene miRNA Labeling kit and a 3D‐Gene Human miRNA Oligo Chip (Toray Industries, Inc., Tokyo, Japan) designed to detect 2562 miRNA sequences registered in miRBase release 21 (http://www.mirbase.org/). miRNA expression data were normalized by using the following four steps: (i) calculation of mean and standard deviation (SD) by using a set of pre-selected negative control signals (background signals) from which the top and bottom 5% of values had been removed; (ii) replacement of signal values greater than the mean + 2 SD of the background signals with log2(signal−mean) and labeling as effective signals; (iii) replacement of the remaining signal values with the minimum of the effective signals−0.1 and (iv) replacement of undetected signal values with the minimum signal of each miRNA. Finally, we used a set of pre-selected internal control miRNAs (miR-149-3p, miR-2861 and miR-4463), which we had found were stably expressed in more than 500 serum samples, to normalize the signals across different microarrays. Each miRNA signal value was standardized by using its ratio to the average signal of three internal control miRNA signals (Figure 1) (26).

### Identification of miRNA-related *cis*- and *trans*-eQTLs

*c* *is*-miR-eQTLs were defined as genetic variants located within 1 Mb upstream or downstream of the mature miRNA sequence, and *trans*-miR-eQTLs were defined as genetic variants located beyond 1 Mb upstream or downstream of the mature miRNA sequence or on another chromosome. The genomic locations of mature miRNA sequences were retrieved from the miRBase database (V22, http://www.mirbase.org/ftp.shtml) (27). We examined associations between the expression of each miRNA and genetic variants by using linear regression, adjusting for age, sex and the top three principal components (PC1, PC2 and PC3); this was implemented by using the eQTL analysis tool Matrix eQTL (28). Matrix eQTL calculates the false discovery rate (FDR) by using expression–variant pairs that have passed a pre-selected significance threshold (‘pvOutputThreshold’). We set the threshold to 0.01 and identified *cis*- and *trans*-miR-eQTLs at an FDR <0.05. These miR-eQTLs were independently identified by using each of the six dementia types, as well as in all subjects (Figure 1).

## Database content and description

### Database content and characteristics of miR-eQTLs

**Figure 2.**
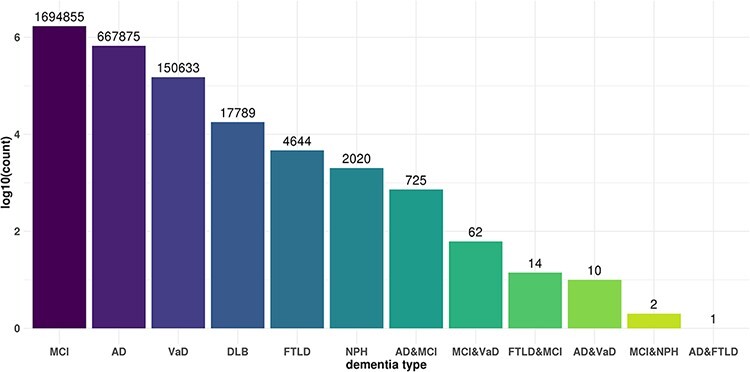
Commonality and specificity of miR-eQTLs among dementia types. X axis shows dementia types and Y axis shows the total numbers of *cis*-miR-eQTLs and *trans*-miR-eQTLs at an FDR <0.05 for each dementia type or combination of dementia types.

We independently analyzed *cis*- and *trans*-miR-eQTLs in the six dementia types and all subjects. We identified a total of 2487 *cis*-miR-eQTLs and 3 155 773 *trans*-miR-eQTLs at an FDR <0.05 in six dementia types and all samples, including those from patients with other types of dementia. We identified 542 *cis*- and 668 069 *trans*-miR-eQTLs in AD, 1 *cis*- and 17 788 *trans*-eQTLs in DLB, 96 *cis*- and 150 609 *trans*-eQTLs in VaD, 4659 *trans*-eQTLs in FTLD, 2022 *trans*-eQTLs in NPH, *1326* cis- and 1 694 332 *trans*-eQTLs in MCI, and 522 *cis*- and 618 294 *trans*-eQTLs in all subjects (Table 1). We further examined the commonality and specificity of miR-eQTLs among dementia types (Figure 2). Compared with the ncRNA-eQTL (19), JAMIR-eQTL detected more trans-eQTLs than cis-eQTLs. The difference could be due to their target ncRNAs. While ncRNA-eQTL targets long non-coding RNAs (lncRNAs), JAMIR-eQTL targets comprehensive miRNAs. lncRNAs are more than 200 nucleotides in length, but miRNAs are far smaller, ∼22 nucleotides in length.

**Table 1.** Summary of samples and numbers of *cis*- and *trans*-miR-eQTLs

		*cis*-			*trans*-		
Dementia type	No. of samples	genotypes	miRNAs	eQTLs	genotypes	miRNAs	eQTLs
AD	1314	535	125	542	458 247	2576	668 069
DLB	134	1	1	1	15 226	602	17 788
VaD	69	96	19	96	33 684	1828	150 609
FTLD	31	0	0	0	3464	54	4659
NPH	39	0	0	0	1875	46	2022
MCI	504	1107	319	1326	332 791	2578	1 694 332
ALL	3448	498	120	522	411 288	2578	618 294

### Quick browsing and searching

**Figure 3.**
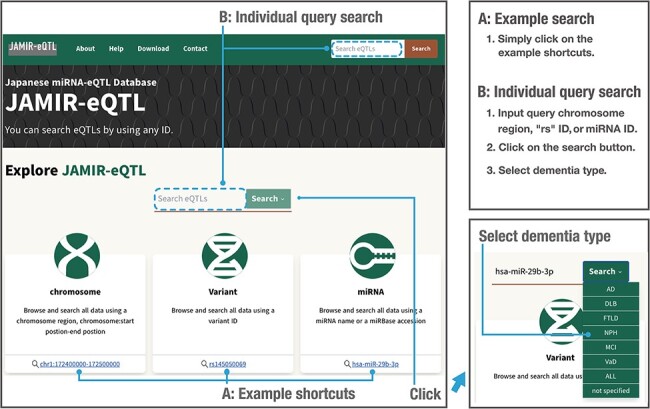
Example and individual query searches in the JAMIR-eQTL database.

To enable data searching and downloading, we developed JAMIR-eQTL, a user-friendly database including comprehensive miRNA-related *cis*-eQTLs and *trans*-eQTLs. The JAMIR-eQTL database has a quick search window in which users can select example shortcuts (Figure 3, A: Example search) or input their genetic variants of interest, miRNAs or genomic locations (Figure 3, B: Individual query search). Variants are searched via refSNP (rs) numbers (e.g. rs145050069), and miRNAs are searched via mRNA names (e.g. hsa-miR-29b-3p) or accession numbers (e.g. MIMAT0000100). Genomic locations are searched via chromosomal start and end positions (e.g. chr1:172400000–172500000); all miR-eQTLs associated with variants included in that range are reported. Users can also select the dementia types that they want to search. Types are composed of AD, DLB, VaD, FTLD, NPH, MCI, ALL and Not specified (six dementia types + ALL). Users can investigate not only the biological correlations between genetic variants of interest and miRNA expressions but also commonality and specificity of the variants among dementia types. Also, the JAMIR-eQTL database can be used to check individual miR-eQTL associations and will be used for validation in large-scale analyses.

### Displaying the database contents

**Figure 4.**
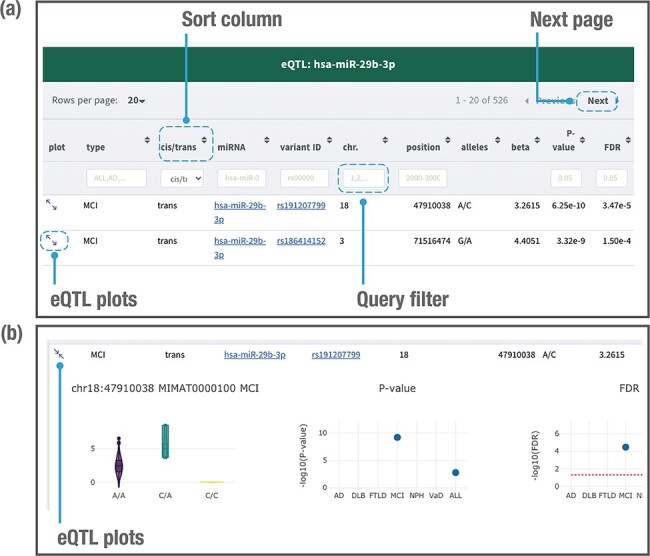
Example of a page displaying search results. The JAMIR-eQTL database returns a table of related miR-eQTL records (a). All columns can be sorted, and records without variant alleles and beta values can be filtered. Detailed information on each eQTL plot can be displayed by clicking on the plot column link (b). A vector diagram of a boxplot displaying the association between variant genotypes and miRNA expression is presented, along with *P*-values and FDRs among the six dementia types and ALL.

When users search by using a genetic variant, miRNA or genomic location of interest, the database returns a table of related miR-eQTL records, consisting of dementia types, *cis*-/trans-miR-eQTLs, miRNA names, ‘rs’ IDs, chromosomes, chromosome positions, variant alleles, beta values (effect sizes of variant on miRNA expression), *P*-values and FDRs (Figure 4a). All columns can be sorted, and records without variant alleles or beta values can be filtered. Detailed information for each eQTL plot can be displayed by clicking on the plot column (Figure 4b). A vector diagram of a boxplot is provided to display the association between variant genotypes and miRNA expression. As the information includes *P*-values and FDRs among the six dementia types and ALL, users can further find out the dementia types between which the miR-eQTLs of interest are shared. Users can also export all plots in PNG (Portable Network Graphics) format.

**Figure 5.**
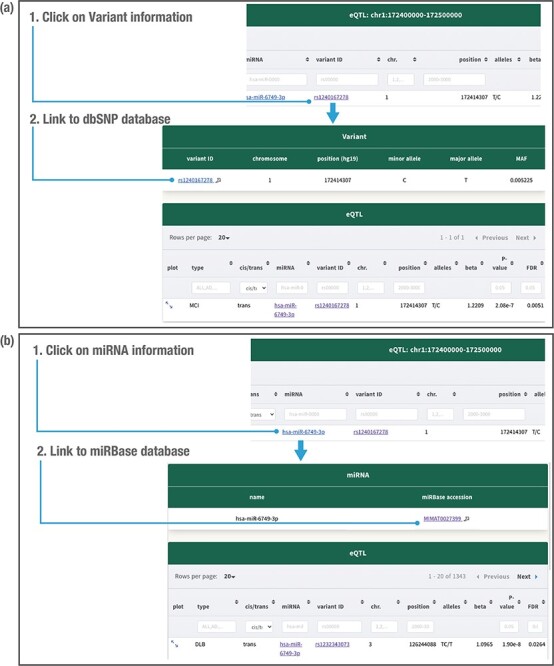
Detailed variant and miRNA information. Clicking on the variant related to the miR-eQTL provides the variant minor allele, major allele and minor allele frequency (MAF) in the JAMIR-eQTL database (a). Clicking on the miRNA provides the microRNA ID and accession number (b). The variant information further links to the public database dbSNP, whereas the miRNA information links to the public database miRbase.

Clicking on the variant related to the miR-eQTL provides the minor allele, major allele and minor allele frequency in our database (Figure 5a). Clicking on the miRNA provides the mRNA ID and accession number (Figure 5b). The variant information is further linked to the public database dbSNP (29), whereas the miRNA information is linked to the public database miRBase (27) (Figure 5a and b). Users can download all miR-eQTL results at FDR < 0.05 for each dementia type from the ‘Download’ section. The ‘About’ page provides summary information for the data collection, processing and results. JAMIR-eQTL welcomes all feedback through email to the address provided on the ‘Contact’ page. We have tested the database on multiple web browsers, including Chrome, Firefox, Windows Edge and Safari.

## Future development

Only one ncRNA-related eQTL database, namely ncRNA-eQTL (19), has recently been developed, for cancer types. The JAMIR-eQTL database is the first miR-eQTL database designed for dementia types. These comprehensive miR-eQTLs are attracting a lot of attention from many researchers focusing on dementia, and there is no doubt that this database is a powerful research tool for studying potential therapeutic mechanisms for dementia. The NCGG Biobank aims to provide a resource for the investigation of the large-scale genetic, environmental and lifestyle determinants of a wide range of aging-associated diseases. Every year, >1000 individuals are enrolled in the Biobank, which had reached 20 000 enrollments as of early 2021. We believe that the JAMIR-eQTL database will continue to increase in size and will be useful to dementia research in the future.

## Implementation

The JAMIR-eQTL database was constructed by using Golang with a PostgreSQL backend-database. A Nuxt.js application was used to build the front-end interface.

## Accessibility

JAMIR-eQTL is freely available to all users without restriction at https://www.jamir-eqtl.org/.

## References

[1] Liu H.S. and Xiao H.S. (2014) MicroRNAs as potential biomarkers for gastric cancer. *World J. Gastroenterol.*, 20, 12007–12017. 25232237 10.3748/wjg.v20.i34.12007 PMC4161788

[2] Qi J. , Wang J., Katayama H. et al. (2013) Circulating microRNAs (cmiRNAs) as novel potential biomarkers for hepatocellular carcinoma. *Neoplasma*, 60, 135–142. 23259781 10.4149/neo_2013_018 PMC3869230

[3] Ren J. , Zhang J., Xu N. et al. (2013) Signature of circulating microRNAs as potential biomarkers in vulnerable coronary artery disease. *PLoS One*, 8, e80738. 10.1371/journal.pone.0080738 PMC3855151 24339880

[4] Denk J. , Boelmans K., Siegismund C. et al. (2015) MicroRNA profiling of CSF reveals potential biomarkers to detect Alzheimer’s disease. *PLoS One*, 10, e0126423. 10.1371/journal.pone.0126423 PMC4439119 25992776

[5] Shigemizu D. , Akiyama S., Higaki S. et al. (2020) Prognosis prediction model for conversion from mild cognitive impairment to Alzheimer’s disease created by integrative analysis of multi-omics data. *Alzheimers Res. Ther.*, 12, 145. 10.1186/s13195-020-00716-0 PMC7656734 33172501

[6] Berezikov E. , Cuppen E. and Plasterk R.H. (2006) Approaches to microRNA discovery. *Nat. Genet.*, 38, S2–S7. 16736019 10.1038/ng1794

[7] Landgraf P. , Rusu M., Sheridan R. et al. (2007) A mammalian microRNA expression atlas based on small RNA library sequencing. *Cell*, 129, 1401–1414. 17604727 10.1016/j.cell.2007.04.040 PMC2681231

[8] Konishi H. , Ichikawa D., Arita T. et al. (2016) Microarray technology and its applications for detecting plasma microRNA biomarkers in digestive tract cancers. *Methods Mol. Biol.*, 1368, 99–109. 26614071 10.1007/978-1-4939-3136-1_8

[9] Krutzfeldt J. , Poy M.N. and Stoffel M. (2006) Strategies to determine the biological function of microRNAs. *Nat. Genet.*, 38, S14–S19. 16736018 10.1038/ng1799

[10] Yengo L. , Sidorenko J., Kemper K.E. et al. (2018) Meta-analysis of genome-wide association studies for height and body mass index in approximately 700000 individuals of European ancestry. *Hum. Mol. Genet.*, 27, 3641–3649. 30124842 10.1093/hmg/ddy271 PMC6488973

[11] Akiyama M. , Okada Y., Kanai M. et al. (2017) Genome-wide association study identifies 112 new loci for body mass index in the Japanese population. *Nat. Genet.*, 49, 1458–1467. 28892062 10.1038/ng.3951

[12] Shigemizu D. , Mitsumori R., Akiyama S. et al. (2021) Ethnic and trans-ethnic genome-wide association studies identify new loci influencing Japanese Alzheimer’s disease risk. *Transl. Psychiatry*, 11, 151. 10.1038/s41398-021-01272-3 PMC7925686 33654092

[13] Conti D.V. , Darst B.F., Moss L.C. et al. (2021) Trans-ancestry genome-wide association meta-analysis of prostate cancer identifies new susceptibility loci and informs genetic risk prediction. *Nat. Genet.*, 53, 65–75. 33398198 10.1038/s41588-020-00748-0 PMC8148035

[14] Cano-Gamez E. and Trynka G. (2020) From GWAS to function: using functional genomics to identify the mechanisms underlying complex diseases. *Front Genet.*, 11, 424. 10.3389/fgene.2020.00424 PMC7237642 32477401

[15] Musunuru K. , Strong A., Frank-Kamenetsky M. et al. (2010) From noncoding variant to phenotype via SORT1 at the 1p13 cholesterol locus. *Nature*, 466, 714–719. 20686566 10.1038/nature09266 PMC3062476

[16] Claussnitzer M. , Dankel S.N., Kim K.H. et al. (2015) FTO obesity variant circuitry and adipocyte browning in humans. *N. Engl. J. Med.*, 373, 895–907. 26287746 10.1056/NEJMoa1502214 PMC4959911

[17] Liang L. , Morar N., Dixon A.L. et al. (2013) A cross-platform analysis of 14,177 expression quantitative trait loci derived from lymphoblastoid cell lines. *Genome Res.*, 23, 716–726. 23345460 10.1101/gr.142521.112 PMC3613588

[18] Consortium G.T. (2015) Human genomics. The Genotype-Tissue Expression (GTEx) pilot analysis: multitissue gene regulation in humans. *Science*, 348, 648–660. 25954001 10.1126/science.1262110 PMC4547484

[19] Li J. , Xue Y., Amin M.T. et al. (2020) ncRNA-eQTL: a database to systematically evaluate the effects of SNPs on non-coding RNA expression across cancer types. *Nucleic Acids Res.*, 48, D956–D963. 31410488 10.1093/nar/gkz711 PMC6943077

[20] Huan T. , Rong J., Liu C. et al. (2015) Genome-wide identification of microRNA expression quantitative trait loci. *Nat. Commun.*, 6, 6601. 10.1038/ncomms7601 PMC4369777 25791433

[21] Yue M. , Zhou D., Zhi H. et al. (2018) MSDD: a manually curated database of experimentally supported associations among miRNAs, SNPs and human diseases. *Nucleic Acids Res.*, 46, D181–D185. 29106642 10.1093/nar/gkx1035 PMC5753252

[22] Shigemizu D. , Akiyama S., Asanomi Y. et al. (2019) Risk prediction models for dementia constructed by supervised principal component analysis using miRNA expression data. *Commun. Biol.*, 2, 77. 10.1038/s42003-019-0324-7 PMC6389908 30820472

[23] Kawai Y. , Mimori T., Kojima K. et al. (2015) Japonica array: improved genotype imputation by designing a population-specific SNP array with 1070 Japanese individuals. *J. Hum. Genet.*, 60, 581–587. 26108142 10.1038/jhg.2015.68 PMC4635170

[24] Howie B.N. , Donnelly P. and Marchini J. (2009) A flexible and accurate genotype imputation method for the next generation of genome-wide association studies. *PLoS Genet.*, 5, e1000529. 10.1371/journal.pgen.1000529 PMC2689936 19543373

[25] Purcell S. , Neale B., Todd-Brown K. et al. (2007) PLINK: a tool set for whole-genome association and population-based linkage analyses. *Am. J. Hum. Genet.*, 81, 559–575. 17701901 10.1086/519795 PMC1950838

[26] Shimomura A. , Shiino S., Kawauchi J. et al. (2016) Novel combination of serum microRNA for detecting breast cancer in the early stage. *Cancer Sci.*, 107, 326–334. 26749252 10.1111/cas.12880 PMC4814263

[27] Kozomara A. , Birgaoanu M. and Griffiths-Jones S. (2019) miRBase: from microRNA sequences to function. *Nucleic Acids Res.*, 47, D155–D162. 30423142 10.1093/nar/gky1141 PMC6323917

[28] Shabalin A.A. (2012) Matrix eQTL: ultra fast eQTL analysis via large matrix operations. *Bioinformatics*, 28, 1353–1358. 22492648 10.1093/bioinformatics/bts163 PMC3348564

[29] Smigielski E.M. , Sirotkin K., Ward M. et al. (2000) dbSNP: a database of single nucleotide polymorphisms. *Nucleic Acids Res.*, 28, 352–355. 10592272 10.1093/nar/28.1.352 PMC102496

